# Factors influencing the occurrence of biliary stricture above the confluence in major bile ducts injuries: Analysis of a case series

**DOI:** 10.1016/j.amsu.2020.07.032

**Published:** 2020-07-22

**Authors:** Anisse Tidjane, Nabil Boudjenan Serradj, Nacim Ikhlef, Noureddine Benmaarouf, Benali Tabeti

**Affiliations:** Department of Hepatobiliary Surgery and Liver Transplantation, EHU-1st November 1954, Department of Medicine, University of Oran 1, Oran, Algeria

**Keywords:** Bile duct injury, Biliary stricture, Laparoscopy, Cholecystectomy, Surgery, North Africa

## Abstract

**Background:**

bile duct injury is a complication that occurs mainly after cholecystectomy. Outcomes of biliary repair surgery are worse when the stricture level is above the biliary confluence.

**Method:**

A single centred retrospective study was carried out on patients operated in our department for biliary stricture after a major bile duct injury over the period from January 2010 to May 2018. Only patients operated for biliary stricture were included. This study aimed to determine the independent factors influencing the occurrence of a stricture above de biliary confluence. Univariate and multivariate binary regression was used for data analysis.

**Results:**

Fifty-three patients were included, they were 43 women and 10 men, sex-ratio was 0.23. Thirty-one patients had Grade E3–E4-E5 stricture (58,5%), and patients who had a failure of a previous repair surgery accounted for 36% (n = 19) of our patients.

After univariate and multivariate analysis, only laparoscopic cholecystectomy (OR = 7.58, CI = [1.47–38, 91], P = 0.015) and failure of anterior biliary repair surgery (OR = 7, 12, CI = [1.29–39.42], P = 0.025) were independent factors associated with more frequent occurrence of biliary strictures above the confluence.

**Conclusion:**

Failure of biliary repair surgery makes the pre-existing biliary stricture progress and compromises subsequent surgery's outcomes. It is important to refer all cases of bile duct injury to specialized centers to increase the chances of success of the first biliary repair surgery.

## Highlights

• We conducted a binary regression analysis of a series of 53 cases of biliary stricture secondary to bile duct injury.
• Bile duct injuries caused during laparoscopic cholecystectomy are associated with the more strictures above the confluence.
• Failure of an anterior biliary repair surgery is associated with more strictures above de confluence.

## 1 Introduction

Operative bile duct injury (OBDI) is a complication that occurs usually during cholecystectomy, either by laparoscopic or open surgery, but can also occur during other upper gastrointestinal surgeries [1]. Even after performing systematic intraoperative cholangiography, diagnosis is often made postoperatively [2]. The main modes of postoperative diagnosis of OBDI are intraperitoneal biliary collection, biliary fistula or biliary stricture [3].

Major OBDI has as a consequence interruption of total bile flow or in two or more sectors of the liver. Therefore, this definition includes injuries causing interruption of bile flow in the main bile duct, in the right hepatic bile duct or the left hepatic bile duct [4]. Thus, injuries involving the sectoral bile ducts are excluded.

After surgical management of major OBDI, several factors seem to influence patient outcomes, among these factors we note the level of biliary stricture. Biliary strictures above the convergence (Hi-S) seem to have a worse prognosis after performing biliary repair surgery than lower-level biliary strictures (Lo-S). Biliary stricture level seems to be an independent factor influencing outcomes after performing biliary repair surgery [5,6].

Given the prognosis of this Hi-S, it is important to recognize factors that can influence their happening.

## 2 Materiel and methods

A retrospective data analysis was performed including all patients admitted for Major OBDI evolving to a biliary stricture in the department of hepatobiliary surgery and liver transplantation of EHU-1st November 1945 , Oran (a tertiary center specialized in hepatobiliary surgery located in western Algeria). The inclusion period sprawled from January 2010 to May 2018. Excluded cases were patients who did not develop stricture and type, A, B, C and D biliary injuries according to the Strasberg classification, so only type E injuries in stricture were included [7].

**Fig. 1:**
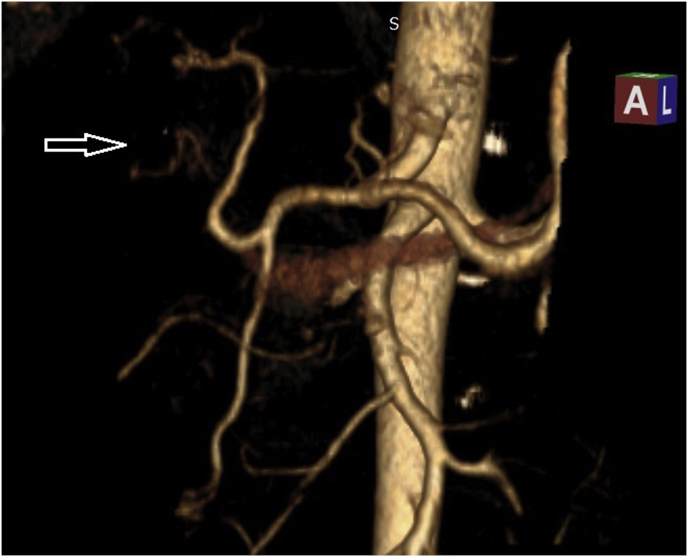
Ct-scan with 3D arterial reconstruction, performed before biliary reconstruction surgery, Arrow: Right arterial obstruction with partial supplementation through the hilar shunt.

**Fig. 2:**
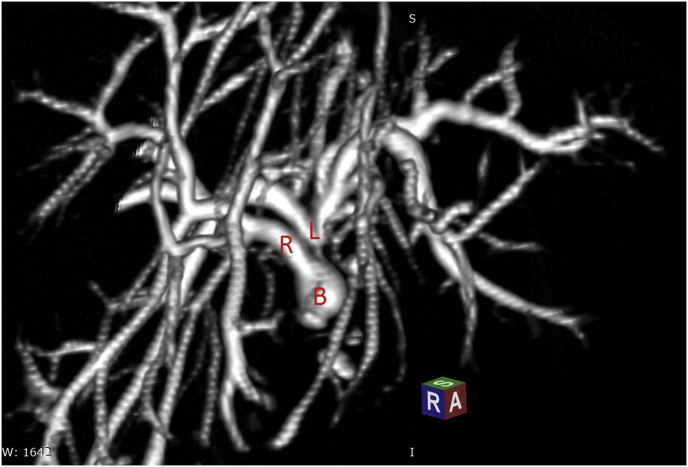
MRI with 3D reconstruction of a biliary stricture, B: Biloma , L = Left hepatic bile duct, R: Right hepatic bile duct.

Our department specialized in hepatobiliary surgery admits patients from different regions of Algeria, often by transfer from other health structures or after specialized consultation and scheduled admission. Patients admitted for OBDI have a standard preoperative evaluation, an evaluation of the hepatic function and anatomical evaluation of injuries with the systematic realization of an MRI and a CT-scan with portal and arterial vascular reconstruction (Fig. 1), (Fig. 2). We are convinced that the best time to perform biliary repair surgery in case of late discovery of the OBDI (after 48 h) is at least 6 weeks, a period often necessary for the regression of inflammatory and infectious phenomena. Biliary repair consists of a bilio-enteric anastomosis on a Y excluded jejunal loop of 75 cm length. The bilio-enteric anastomosis was made according to the technique described by Hepp [8] or after confection of new biliary confluence and in some cases by performing two or more separated bilio-enteric anastomosis [8]. The definition of biliary stricture level is based on the results of MRI performed at the latest a week before surgery with three-dimensional reconstruction. Final confirmation is made during biliary repair surgery by performing intraoperative cholangiography.

Included patients were divided into two groups: patients with Strasberg type E1 and E2 "Lo-S" for the first group and patients with type E3, E4 and E5 "Hi-S" for the second group. This study aimed to determine the independent factors influencing the occurrence of a Hi-S.

Association between the Hi-S (Variable to be explained) and the various factors related to the patient, his surgery, his postoperative events and associated vascular lesions (Values to be explained) were analyzed using binary regression. Data were analyzed using IBM software, SPSS V20. A univariate and multivariate binary regression analysis was carried out in search of the influence of different parameters on the occurrence of a Hi-S. All factors having a P < 0.20 in univariate analysis were included in a multivariate analysis, Odds ratio was calculated with a confidence interval of 95%, a probability value P ≤ 0.05 was considered to be statistically significant.

This work is written according to PROCESS criteria [9].

This study was retrospectively registered on http://www.ClinicalTrials.gov (NCT 04286763). The ethical approval of this research was obtained from the scientific council of the department.

## 3 Results

During these 97 months, 53 patients underwent surgical repair for biliary stricture following a major OBDI. There were 10 males and 43 females, sex-ratio was 0,23. The average age of our patients was 44.3 years with extremes ranging from 20 to 77 years. Delay of reference after OBDI was 07 months with extremes ranging from 2 to 171 months. In all cases injury was caused by surgeons exercising in other surgical departments. 19 patients (35.8%) underwent biliary repair surgery performed in other health structure before their referral to our tertiary center, from these: 17 patients (32.1%) had one biliary repair surgery with poor results, one patient (1.9%) had two surgeries and another patient (1.9%) underwent three surgeries before reference.

The OBDI in our cases was caused during a laparoscopic cholecystectomy in 52.8% of cases (n = 28), an open cholecystectomy in 39.6% of cases (n = 21), or following an exploration of the main bile duct during cholecystectomy in two patients that represents 3.8% of cases (n = 2) and in two other patients following surgery for a hydatid cyst of the liver located near the hilum 3.8% (n = 2). Among our patients, only 71.7% (n = 38) had useable information's concerning the course of surgery that caused the injury. From these 38 patients, 50% (n = 19) were operated in cholecystitis.

Biliary stricture was classified according to Strasberg classification, type E1 represents 3.8% of our patients (n = 2), type E2 37.7% (n = 20), type E3 37.7% (n = 20), type E4 17% (n = 9) and type E5 3.8% (n = 2). Hi-S was present in 58.5% of patients (n = 31). Only 9.5% (n = 5) of our cases had an associated vascular lesion on the right branch of the hepatic artery, this injury was suspected preoperatively after performing an Angio-CT and confirmed during biliary repair surgery (Table 1).

**Table 1:** Description of the study population.

	Number	%
Age years (Average ± Deviation)	44,30± 13,82	
Sex (AP=53):		
- Female	43	81,1%
- Male	10	18,9%
Comorbidities (AP=53):		
- Diabetes:	11	20,75%
- Hypertension:	8	15,09%
- Heart disease:	7	13,20%
- Dysthyroidism:	3	5,66%
- Obstructive pulmonary disease:	2	3,77%
- Other comorbidities:	4	7,54%
Type of surgery (AP:53)		
- Cholecystectomy ∗ *Laparoscopic cholecystectomy* ∗ Open cholecystectomy	49	92,5%
	*28*	*52,9%*
	*21*	*39,6%*
- Main bile duct exploration	2	3,8%
- Liver hydatic cyst surgery	2	3,8%
Structure were the injury occurred (AP=53)		
- Teaching/Academic hospital:	03	5,7%
- Public hospital:	36	67,9%
- Private structure:	14	26,4%
Immediate recognition of bile duct injury (AP=43)		
- Immediate recognition	13	24,53%
- Postoperative recognition	40	75,47%
Transfer delay (month): (Average ± Deviation)	13,57 ± 24,8	
Information regarding surgery (AP=53)		
- Not available or poor	15	28,3%
- Available	38	71,7%
Difficulty during initial surgery (AP=53)		
- Cholecystitis.	19	35,8%
- Scleroatrophic gallbladder.	5	9,4%
- Hepatic pedicle inflammation.	9	17%
- Adhesions	16	30,2%
- Biliary fistula.	1	1,9%
- Bleeding	10	18,9%
- No difficulty	5	9,4%
- Not available or poor	15	28,3%
Anterior reconstruction biliary surgery ( AP=53)		
- Yes - *One surgery* - *Two surgery’s* - *Three surgery’s*	19	35,8%
	*17*	*32,1%*
	*1*	*1,9%*
	*1*	*1,9%*
- No	34	64,2%
Repartition according to Strasberg Classification (AP=53)		
- E1	2	3,8%
- E2	20	37,7%
- E3	20	37,7%
- E4	9	17%
- E5	2	3,8%
Level of biliary stricture (AP=53)		
- Low level of stricture (E1,E2)	22	41,5%
- High level of stricture (E3,E4,E5)	31	58,5%
Association with vascular injury (AP=53)		
- Arterial injury:	5	9,4%
- Portal injury	0	0%
- No vascular associated injury	48	90,6%

Abbreviation: AP: Analyzed population.

A binary regression statistical analysis was carried out to look for the factors which could influence the occurrence of Hi-S, the factors analyzed were: sex, age, open or laparoscopic cholecystectomy, cholecystitis, bleeding during surgery, immediate recognition of OBDI, postoperative biliary peritonitis, delayed transfer to our department, failure of a previous biliary repair surgery and associated vascular injury.

After univariate binary regression analysis, it seems that only laparoscopic cholecystectomy (P = 0.023) and failure of an anterior biliary repair surgery (P = 0.029) influenced the occurrence of more Hi-S (Table 2).

**Table 2:** Univariate binary regression analysis to determine the factors influencing the occurrence of high level biliary stricture.

	Low level biliary stricture (Strasberg E1 and E2)		High level biliary stricture (Strasberg E3,E4 and E5)		Total	P Value
	N	%	N	%		
Age (years):(mean ± deviation)	46,27 ± 12,43		42,90 ± 14,75		44,3 ± 13,81	0,380
Sex (AP = 53):						
Female	18	41,9%	25	58,1%	43	0,914
Male	4	40%	6	60%	10	
Cholecystectomy(AP = 51):						
Laparoscopic cholecystectomy	8	28,6%	20	71,4%	28	0,023*
Open cholecystectomy	14	60,9%	9	39,1%	23	
Cholecystis (AP = 38):						
Yes	10	52,6%	9	47,4%	19	0,193**
No	6	31,6%	13	68,4%	19	
Occurrence of bleeding during surgery (AP = 38):						
Yes	4	40%	6	60%	10	0,875
No	12	42,9%	16	57,1%	28	
Immediate recognition of bile duct injury(AP = 53):						
Yes	3	23,1%	10	76,9%	13	0,131**
No	19	47,5%	21	52,5%	40	
Occurrence of biliary peritonitis (AP = 53)):						
Yes	7	46,67%	8	53,33%	15	0,633
No	15	39,47%	23	60,53%	38	
Association with vascular injury (AP = 53):						
Yes	2	40%	3	60%	5	0,943
No	20	41,7%	28	58,3%	48	
Anterior surgical biliary repair (AP = 53):						
No	18	52,9%	16	47,1%	34	0,029*
Yes	4	21,1%	15	78,9%	19	
Delay of referral (month)						
(mean ± deviation)	11,52 ± 13,68		14,31 ± 30,10		13,57 ± 24,8-	0,634

Abbreviations: N: number of cases, %: percentage, AP: Analyzed population, P: Probability value, *: P < 0,05, **: P < 0,20.

All analyzed factors in univariate analysis and whose P value was less than 0.20 were included in a multivariate binary regression analysis. Thus, the presence of biliary injury during laparoscopic cholecystectomy was associated with more frequent occurrence of Hi-S compared to stricture occurring after open surgery (OR = 7.58, CI = [1.47–38, 91], P = 0.015). Failure of a previous biliary repair surgery was associated with more Hi-S compared to patients who never had biliary repair surgery after OBDI (OR = 7, 12, CI = [ 1.29–39.42], P = 0.025). The presence of cholecystitis during cholecystectomy does not seem to have any influence on the occurrence of more Hi-S (OR = 0.21, CI = [0.03–1.33], P = 0.098). Immediate recognition of OBDI during the first surgery which caused the biliary injury does not also seem to influence the occurrence of more Hi-S (OR = 1.68, CI = [0.21–13.45], P = 0.622) (Table 3).

**Table 3:** Multivariate binary regression analysis to determine the factors influencing the occurrence of high level biliary stricture.

Analyzed variable	OR	CI (95%)		P
Laparoscopic cholecystectomy	7,580	1,476	38,915	0,015 *
Anterior surgical biliary repair	7,122	1,287	39,425	0,025 *
Acute cholecystitis	0,214	0,035	1,327	0,098
Immediate recognition of biliary injury	1,685	0,211	13,448	0,622

Abbreviation: OR:Odds Ratio, CI: confidence interval, P: probability value, *: P<0,05.

## 4 Discussion

The predominance of female gender in different literature series of OBDI is clear, in our series it was 81% of cases, the average age of our patients was between the fourth and fifth decade [10]. Reference delay for patients who had an OBDI varies from a series to another. In the majority of developed countries series, this reference delay does not exceed 3 months, unlike the series from developing countries where these periods seem longer [11,12]. It should be noted that this reference delay is also extended in the series including patients operated after the failure of biliary repair surgery performed in other centers [13,14]. In our series the median of this period was 7 months.

In the majority of tertiary center series, a large number of patients were treated after the failure of biliary repair surgery often performed in non-expert centers. In some cases the repair surgery is performed by the surgeon who caused the OBDI [5,11,13,15]. The results of biliary repair surgery are worse when this surgery is performed by a non-hepatobiliary surgeon [16,17]. In our series, more than a third of our patients had already a biliary repair surgery performed in other surgery departments, and in few patients even several biliary repair surgeries with bad results were performed.

The surgery that caused OBDI in our patients was mainly cholecystectomy. In our series we still count injuries caused during open cholecystectomy. It should be noted that in Algeria, and according to a survey carried out in 2017 on surgical practices during cholecystectomy, 22% of participant surgeons performed open surgery [18]. This explains the large rate of injuries caused by open cholecystectomy in our series. Whereas in the other series, the main cause of OBDI remains laparoscopic cholecystectomy. It should be noted that the laparoscopic approach is not only responsible for increasing incidence of OBDI, but also of its severity and complexity [19].

Among our patients, we also count two cases of OBDI following liver hydatid cyst surgery (LHC), the lesion of the bile ducts was not caused by the progression of the disease but by the curative surgical procedure. LHC is a parasitic disease frequently encountered in rural areas in Algeria like all countries known by sheep farming activities. During our review of literature, no case of OBDI was described following this LHC surgery and the described cases are due to destruction caused by the cyst's growth [20].

The availability of detailed information concerning circumstances of OBDI occurrence was only available for 71.7% of our patients, this lack of information was an obstacle for patient management and statistical analysis. This problem is not detailed in publications, an Iraqi publication reports this problem, this lack of information is probably especially encountered in developing countries [21].

As a factor considered to favor OBDI, cholecystitis had a preponderant place, 50% of OBDI in our patients were provoked during cholecystectomy for cholecystitis [22].

Major part of our patients had Hi-S. Like the majority of tertiary centers series, the most complex biliary strictures are referred to centers specialized in hepatobiliary surgery. This emerges after reviewing different series [12,13,21,23].

We did systematically search for associated vascular lesions in our patients by performing a CT-scan with vascular reconstruction. Our series has a low rate of vascular lesions (9.5% of cases). This is probably because many of our patients were operated under open surgery [24]. We find no other likely explanation for this low rate of associated vascular injury. An association was noted between biliary stricture level and the risk of arterial vascular lesion association, as in the series of Buell et al. that reports an incidence of arterial vascular-biliary lesion at the rate of 8%, 63% and 75% respectively for lesions of types E3, E4, and E5 [25].

After a univariate analysis of factors that may influence Hi-S occurrence, only two factors were retained. Laparoscopic cholecystectomy compared to open cholecystectomy (P = 0.023) and failure of a previous biliary repair surgery compared to patients with no history of biliary repair surgery (P = 0.029). And these same two factors were retained in multivariate analysis as independent factors responsible for Hi-S occurrence after major OBDI.

The occurrence of a more frequent Hi-S in the era of laparoscopic cholecystectomy has been described in the literature, but without any real statistical evidence to consolidate this finding [[25], [26], [27]]. In previous series where OBDI was caused under open cholecystectomy, Lo-S accounted for more than 75% of cases [15], in most recent series including exclusively OBDI caused by laparoscopic cholecystectomy, stricture became Hi-S in more than 60% of cases [12].

Illusion, bi-directional vision and the absence of haptic sensation during laparoscopic cholecystectomy often lead to confusion between the main bile duct and the cystic duct and have as consequence almost total resections of the main bile duct, with injuries carried high above the biliary confluence and more frequently associated arterial vascular lesions of the right hepatic artery [17,[28], [29], [30], [31]].

In our series we noticed that patients who had biliary repair surgery after OBDI tended to have more Hi-S, this would probably be explained by a progression of the stricture which gains in height after each failure of biliary repair surgery and consumes more precious biliary tissue.

Association between Hi-S and vascular lesion is described, it can reach 52% for E4 type lesions, while it does not exceed 18% for types E1 and E2 lesions [17]. However in our series which only counts 5 cases (9.4%) of associated vascular lesions, there seems to be no association between vascular lesion and the occurrence of Lo-S or Hi –S (40% vs 60%, P = 0.943), this negative result can be explained by the fact that our series has a low number of cases of associated vascular and biliary injuries, which does not allow powerful statistical analysis of this variable [32].

Age, gender, cholecystitis, bleeding during cholecystectomy, associated vascular injury, immediate or late recognition of the OBDI, postoperative biliary peritonitis and referral delay to a tertiary center are factors that do not seem to have an influence on the occurrence of Hi-S in our patients after univariate analysis.

### 4.1 Our study has the following weaknesses

- The fact that it was a retrospective study.
- The absence of information related to the course of the surgery which caused OBDI in a large number of patients.

However, our series includes strengths, such as the large number of OBDI caused by open cholecystectomy, which allowed an analysis of the influence of laparoscopic surgery on the occurrence of Hi-S. The report of the only two cases described in the literature of OBDI occurred during HLC surgery, and finally the fact that our study is the only one that interested in factors influencing the occurrence of Hi-S after major OBDI.

## 5 Conclusion

After occurence of a major bile duct injury, each failure of biliary repair surgery increases the risk of biliary stricture progression above the biliary confluence, this risk is multiplied by 7.1 compared to patients who had no anterior biliary repair surgery, hence the interest of referring these patients exclusively to specialized centers where chances for successful first biliary repair surgery are highest.

In the laparoscopic surgery era, if the risk of operative bile duct injury is currently equal to that encountered in the era of an open procedure, it should be noted that major bile duct injuries caused during laparoscopic cholecystectomy tend to affect the biliary confluence. Therefore, it is advisable to take more precautions during this surgery, to adopt the critical view of safety and to resort when necessary to imaging techniques allowing clear visualization of bile ducts. Because bile duct injuries caused by laparoscopic procedures are more complex and higher. However, a prospective milticentric study is necessary to confirm our results.

## Author statement

A.TIDJANE : Study design , data collection & analysis , writing.

N.Boudjenan Serradj : Writing, critical review

N.Ikhlef data collection & analysis , writing.

N.Benmaarouf : Writing, critical review

B.Tabeti Writing, study design critical review.

## Study registration

This study was retrospectively registered on http://www.ClinicalTrials.gov (NCT 04286763).

## Disclosure of funding

None

## Funding

This research did not receive any specific grant from funding agencies in the public, commercial, or not-for-profit sectors.

## Ethical approval

Obtained from the scientific council in charge of scientific watch of all research projects of the department.

## Provenance and peer review

Not commissioned, externally peer reviewed.

## Research registration Unique Identifying number (UIN)

1.Name of the registry:

Factors Influencing Occurrence Of Hilar Biliary Stricture In Case of Bile Duct Injury.

2.Unique Identifying number or registration ID: NCT 04286763.

3.Hyperlink to your specific registration (must be publicly accessible and will be checked):

https://www.clinicaltrials.gov/ct2/show/NCT04286763?term=NCT+04286763&draw=2&rank=1.

## Guarantor

Pr Benali Tabeti.

Head of the department of hepatobiliary surgery and liver transplantation, EHU-1^St^ November 1954, Oran, Algeria.

## Declaration of competing interest

None.
